# Functional Concentrations of BMP4 on Differentiation of Mouse Embryonic Stem Cells to Primordial Germ Cells

**Published:** 2011-09-23

**Authors:** Hatef Ghasemi Hamidabadi, Parichehr Pasbakhsh, Fardin Amidi, Masoud Soleimani, Mehdi Forouzandeh, Aligholi Sobhani

**Affiliations:** 1 Department of Anatomy, Faculty of Medicine, Tehran University of Medical Sciences, Tehran, Iran; 2 Department of Hematology, Faculty of Medical Sciences, Tarbiat Modares University, Tehran, Iran; 3 Department of Biotechnology, Faculty of Medical Sciences, Tarbiat Modares University, Tehran, Iran

**Keywords:** BMP4, CGR8, Optimal Doses, Mvh Positive Cells, PGCs

## Abstract

**Background:**

Bone morphogenetic protein 4 (BMP4) has a significant role in primordial germ cells (PGCs) differentiation from mouse embryonic stem cell (mESC). The aim of this study is to determine the best concentration of BMP4 at a time of two days on differentiation PGCs from mESC.

**Materials and Methods:**

To differentiate PGCs, embryoid bodies (EBs) from mESCs were cultured in concentrations of 0, 5 and 10 ng/ml BMP4 for two days. Germ cell markers *Oct4* (*Pou5f1*), *Stella* (*Dppa3*) and *Mvh* (*Ddx4*) were analyzed by flow cytometry, immunocytochemistry and reverse transcriptase polymerase chain reaction (RT-PCR).

**Results:**

Flow cytometry data demonstrated most *Mvh*-positive cells were observed only in the treated groups. Immunocytochemistry of EBs in the treated groups identified cells positive for *Mvh*. PCR results showed expression of *Oct4* in the control group and treated groups. *Stella* and *Mvh* were expressed only in the treated groups.

**Conclusion:**

Low concentrations of BMP4 during two days had an optimal effect on differentiation of PGCs from mESC.

## Introduction

Introduction Primordial germ cells (PGCs) are derived from the extreme proximal region of the epiblast adjacent to the extra-embryonic ectoderm. Some growth factors have been shown to induce PGCs differentiation from stem cells (1). One such factor is bone morphogenetic protein 4 (BMP4), a member of the transforming growth factor β (TGFβ) superfamily of intercellular signaling proteins which has a notable value in PGCs induction (2-4). In addition, other BMPs (BMP8b and BMP2) have been shown to be of importance for establishing normal numbers of PGCs. Analysis of mice carrying null mutations of the *Bmp4, Bmp7 and Bmp8b* genes has revealed that *Bmp4* null mutants have the most severe defect in germ cell development, with a near-complete absence of PGCs (3). Members of this family signal through heteromeric complexes composed of types I and II serine-threonine kinase receptors. Binding of BMPs to their receptors induces phosphorylation of the BMP-specific Smads (Smad1, Smad5 and Smad8). Upon activation, these Smads bind to Smad4 and translocate from the cytoplasm to the nucleus, where they regulate transcription of BMP target genes (5-7).

BMPs have known pivotal roles in germ cell development and function, in particular BMP4 controls the formation and early proliferation of PGCs. BMP4, which is released from extra-embryonic ectoderm, provides a proper condition for PGCs differentiation from epiblast cells (3, 8-10). The cells are distinguished from surrounding cells by their unique gene expression patterns. The most common genes used for their identification are *Oct4* (*Pou5f1*), *Stella* (*Dppa3*) and *Mvh* (*Ddx4*). These genes are involved in regulation of their migration and differentiation, and in maintaining the pluripotency of PGCs (11-14).

Some reports exist regarding the differentiation of PGCs from embryonic stem cells (ESCs) by inducing formation of embryoid bodies (EBs) and the addition of certain growth factors, such as BMP4, to cultures. Several studies have demonstrated the ability of EBs to support differentiation of germ cells *in vitro* (15-17). Cultured EBs in the presence of BMP4 produce cells which they mimic in an in vivo system of germ cell differentiation (15). After one day in the presence of BMP4, *Mvh*-positive cells are obtained from EBs (18). This *in vitro* system can potentially be used as a research tool to enable a better understanding of germ cell differentiation. *In vitro* germ cell differentiation makes a model for lineage commitment, specification and PGCs that are often extremely difficult to access in vivo (13).

Recently, the development of PGCs derived from epiblast cells using an *in vitro* culture system have been studied by Hayashia et al. Their result demonstrated that epiblast cells differentiated into PGCs upon addition of BMP4 in the culture in a dose dependent manner. The average number of PGCs from epiblast cells cultured with BMP4 gave rise to more PGCs and induced phosphorylation of SMAD proteins more strongly than those seen in cultured epiblast cells with extra-embryonic ectoderm (19, 20). Dudley et al. has reported that PGCs numbers were mediated by BMP signaling (6).

Despite previous reports that have shown which BMP4 in tissue culture acts in a dose dependent manner, few studies have demonstrated the effects of functional doses of BMP4 on PGCs differentiation from mouse embryonic stem cells (mESCs) *in vitro*. Based on this evidence, we have designed an *in vitro* cell culture system for differentiation of PGCs from mESC using doses of 0, 5 and 10 ng/ ml of BMP4.

## Materials and Methods

This project was approved by the Ethics Committee of Tehran University of Medical Sciences. In this study, we utilized concentrations of 0, 5 and 10 ng/ml of BMP4 (R & D System, USA) for a culture period of two days. We designed our control and treatment groups based on the receiving doses of BMP4 as follows: D2B0 for the control group, D2B5 and D2B10 for treatment groups.

### ESC culture

CGR8-GFP mESC, established from strain 129 (a gift from Dr. M. Solimani), was maintained in the absence of feeder cells. CGR8-GFP was cultured on gelatin (0.1% Sigma, USA)-coated 50 ml plastic flasks (Falcon, Becton Dickinson) in knock out dulbecco’s modified eagle medium (DMEM) that contained high glucose and pyruvate (Gibco-Life Technologies, Canada) supplemented with 10% fetal bovine serum (FBS); (Gibco-Life Technologies, Canada, batch no. 12021565), leukemia inhibitory factor (LIF) (1,000 IU/ml; Chemicon, Boronia, Australia), 1% w/w non-essential amino acids (Gibco-Life Technologies, Canada), 0.1 mM β-mercaptoethanol (Sigma, USA) and 1% w/w penicillin/streptomycin (Gibco-Life Technologies, Canada). The cells were incubated at 37°C in 5% CO2. Primary cultures were allowed to reach confluence before cells were lifted, split and replated (21).

### Embryoid bodies

EBs were created using the hanging drop method. Once secondary ESC cultures reached confluence, cells were lifted as described before, washed and resuspended in LIF-free knock out DMEM supplemented with 10% FBS to a concentration of 2000 cells per 20 μl. Twenty micro liter drops of the suspension were placed on the lid of a 10 cm plastic culture dish (Falcon). The lid was turned upside down and placed on the bottom part of dish, which was filled with sterile water, creating hanging drops. The cells were incubated at 37°C in 5% CO2. EBs were cultured for 48 hours before being transferred to differentiation medium for the a period of two days (22).

### Immunocytochemistry

For immunocytochemistry, EB cells in each group were washed with phosphate buffered saline (PBS) at pH 7.4 and fixed in 4% paraformaldehyde (PFA) for 30 minutes at room temperature (RT). Fixed cells were permeabilized with 0.2% triton X-100 for 10 minutes at RT followed by three washes with PBS. To block unspecific binding of the antibody, cells were incubated with 10% goat serum for 30 minutes at RT. Then, cells were incubated with primary antibody *Ddx4*/ *Mvh* (rabbit polyclonal IgG, ab13840, Abcam System, UK) 1:100 in the diluted antibody in 1% bovine serum albumin (BSA) in PBS, overnight at 4°C. The solution was decanted and the cells washed three times in PBS. Further incubation with the secondary antibody phycoerythrin (PE) -conjugated donkey polyclonal secondary antibody to rabbit IgG (ab 7007, Abcam System, UK) was performed for 45 minutes at RT in the dark, then the secondary antibody solution was decanted and cells were washed three times in PBS. Cells with only secondary antibody staining were negative controls. Nuclei were detected by DAPI (Sigma, USA) staining. Images were captured with an Olympus phase contrast microscope (BX51, Olympus, Tokyo, Japan).

### Flow cytometry

EB cells were washed with PBS and treated with trypsin/EDTA for 5 minutes to form single cells. The suspension was collected by centrifuging at 2000 rpm for 5 minutes. For intracellular staining of *Mvh* protein, EB cells were fixed in 1% PFA for 10-15 minutes at 4°C for stabilizing proteins, followed by permeablizing of cells in detergent (0.2% triton-X 100). Fixation/permeablization procedures had to be on ice. The cells were washed by adding 2 ml PBS and centrifuged at 2000 rpm for 5 minutes. The supernatant was discarded and the pellet re-suspended in goat serum for 45 minutes to block nonspecific antibody binding. Cells were labeled with rabbit polyclonal IgG *Mvh* antibody (Abcam System, UK) overnight at 4°C in the dark, and they were washed three times and centrifuged at 2000 rpm for 5 minutes, and resuspended in ice cold PBS. PE-conjugated donkey polyclonal to rabbit IgG (Abcam System, UK) was used as a secondary antibody and the cells were incubated for 30 minutes at 4°C. Analysis was performed as soon as possible using a BD FACS Caliber (Becton Dickinson, San Jose, CA, USA) and FlowJo software (WinMDI 2.9, J. Trotter).

### RNA extraction and PCR

EB cells were collected at the designated periods of the culture by aspiration into an Eppendorf tube, centrifuged, and the supernatant discarded. Total RNA was extracted using the qiazol lysis reagent (Qiagen) according to the manufacturer’s instructions. Total RNA was quantified and 5 μg was used for cDNA synthesis using random primers (Fermentas) under standard conditions. RT-PCR amplifications were conducted for 3 minutes at 95°C (95°C, 30 seconds; 60°C, 45 seconds; and 72°C, 45 seconds) for 40 cycles and 72°C for 7 minutes for the final extension. Primer sequences are shown in table 1.

**Table 1:** Quantitative RT-PCR primer sequences


Gene	Primer (forward/reverse)	Significance

	5'-CTTCTTGGGTATGGAATCCTG -3' 5'-GTGTTGGCATAGAGGTCTTTAC. -3'	Internal control
**Oct4**	5'-GTTCTCTTTGGAAAGGTGTTC-3' 5'-GCATATCTCCTGAAGGTTCTC -3'	Pluripotency marker
**Stella**	5'- TGAAGAGGACGCTTTGGA-3' 5'- CTTTCAGCACCGACAACA -3'	Germ cell marker
**Mvh**	5'-CGGAGAGGAACCTGAAGC -3' 5'- CGCCAATATCTGATGAAGC -3'	Germ cell marker


### Data analysis

Quantitative data were expressed as means ± SEM from at least three experiments. One-way ANOVA was used for statistical analysis with p<0.05 considered significant.

## Results

In this study, we assayed PGCs formation from CGR8-GFP and *Mvh*-positive expression by flow cytometry.

**Fig 1:**
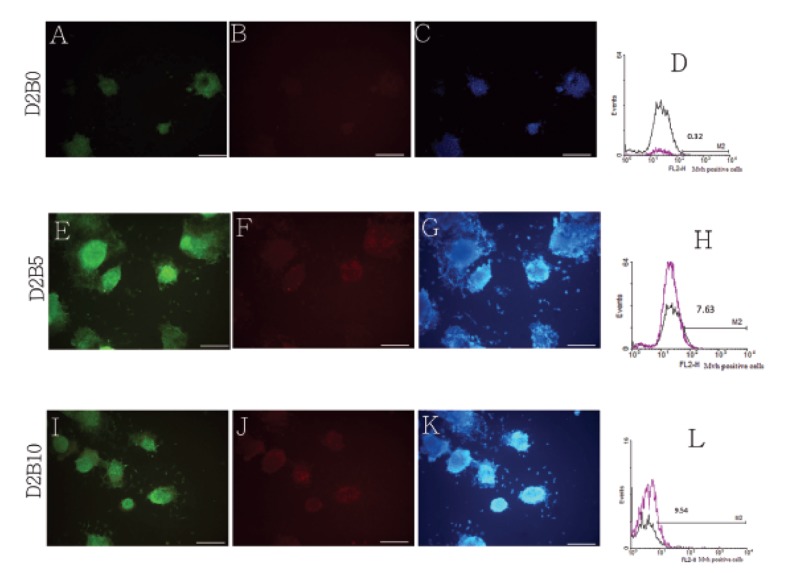
Immunocytochemistry staining of EB cells for fluorescent cells. First panel: D2B0 (A), D2B5 (E) and D2B10 (I). The second panel shows ICC staining of EB cells for Mvh: D2B0 (B), D2B5 (F) and D2B10 (J). The third panel shows DAPI staining; D2B0 (C), D2B5 (G) and D2B10 (K). Scale bars: 50 μm. Analysis by flow cytometry indicated a small population of Mvh-positive cells in mESC. Proportion of PGCs derived from mESC in D2B0 (0.32%) (D), D2B5 (7.63%) (H) and D2B10 (9.54%) groups (L), Negative controls are shown in black.

In the control (undifferentiated) group less than 0.5% of the population was positive for *Mvh* (Fig 1D). The population of *Mvh*-positive cells in the D2B5 group was 7.63% whereas it was 9.54% in the D2B10 group (Fig 1H, L). Additionally, statistical analysis demonstrated no significant differences between the groups.

To recognize other specific characteristics of PGCs derived from mESC, we applied immunocytochemistry. Among the entire marker that was used to distinguish between mESCs and PGCs, *Mvh* was the best marker as this protein was observed in PGCs. However, we did not detect this protein in non-differentiated mESCs (Fig 1B). It was important that *Mvh*-positive appeared as ring-like structures at the edge of the EBs in the treated groups (Fig 1F, J). They looked exactly like the sex cord in an embryo gonad.

**Fig 2:**
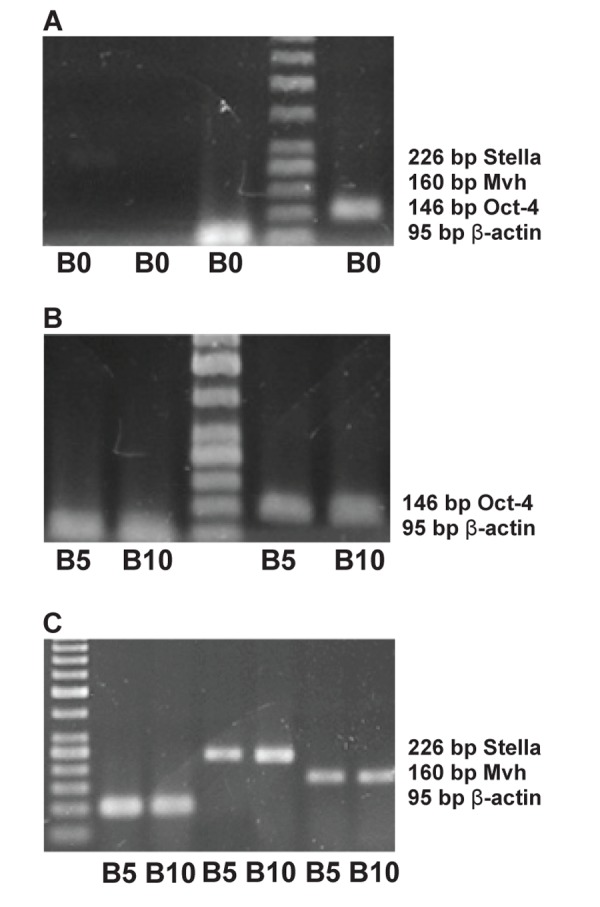
Reverse transcription–polymerase chain reaction for the identification of germ cell markers. (A) RNA was prepared from the control group (D2B0) for Oct4, Stella and Mvh. (B) RNA was prepared from treated groups (D2B5 and D2B10) for Oct4. RNA was prepared from treated groups (D2B5 and D2B10) for Stella and Mvh. β-actin served as an internal mRNA control.

Expressions of *Oct4* (*Pou5f1*), *Stella* (*Dppa3*) and *Mvh* (*Ddx4*) were studied by RT-PCR analysis. By gel electrophoresis, expression of gene *Oct4*, considered as a marker of pluripotency, was observed in the control (D2B0) and treated groups (D2B5 and D2B10) (Fig 2A, B).

Another gene, *Stella*, is a germ cell marker during early development. The results of gel electrophoresis revealed that *Stella* on the second day at BMP4 concentrations of 5 and 10 ng/ml was expressed, but expression of this gene in the control group (D2B0) was not observed (Fig 2A, C). *Mvh*, as a specific germ cell marker, was expressed on the second day with BMP4 concentrations of 5 and 10 ng/ml, but not in the control group (Fig 2A,C).

## Discussion

BMP4, a mesoderm inducer, plays an important role in PGC generation in vivo (1). In this study, we applied BMP4 to our differentiation model. This study demonstrated that *Mvh*-positive cells could be differentiated from mESC *in vitro*. Recently, many researchers verified the effect of different factors on differentiation of human ESCs (hESCs) and mESCs to PGCs (6, 15, 23). However, there is not enough information about the effect of a functional concentration of BMP4 on differentiation of stem cells to PGCs under *in vitro* conditions. We have attempted to demonstrate the impact of different concentrations of BMP4 after two days of culture.

An EB differentiation process was firstly adopted because a 3-d culture would more closely mimic conditions in situ than a monolayer culture (24). Immunocytochemistry results for EBs showed that *Mvh*-positive cells appeared as a cluster at the edge of EBs, which was observed at BMP4 concentrations of 5 and 10 ng/ml on the second day. This confirmed the results of other studies (15, 18, 24, 25). Additionally, we showed that a population of *Mvh*-positive cells were generated from mESCs. In order to confirm this finding, flow cytometry was utilized to distinguish the effect of different concentrations of BMP4 over a period of two days. The *Mvh* protein, a germ cell specific marker in mice, was observed in the treatment groups. Few *Mvh*-positive cells, however, were observed in the control group.

The percent of *Mvh*-positive population in the D2B0 group was less than 0.5%. This result demonstrated the spontaneous presence of *Mvh*-positive cells in EBs, even without the addition of BMP4. Regarding this, Clark et al. (15) have mentioned that human ES cells can attain germ cells to the stage of Vasa (*Mvh*) expression by spontaneous differentiation *in vitro*. Some studies that differentiated ESCs into germ cells used undefined culture conditions that included FBS which might have affected differentiation via BMP activity. These results have suggested that the inherent BMP activity of these systems was sufficient to cause differentiation into germ-like cells. Initial PGCs development can occur spontaneously for ESCs in EBs (2, 15, 26). It appears that embryonic cell cultures contain different factors necessary for differentiation of ESCs to PGCs, although it is not sufficient.

The ability of exogenous BMP4 to potentiate germ cell differentiation has been previously evaluated. In this study we demonstrated that BMP4 was sufficient to form PGCs in an *in vitro* culture during two days. It seemed that the concentration of BMP4 affected the percent of *Mvh*-positive cells. The populations of positive *Mvh* cells in the D2B10 and D2B5 groups were approximately 10 % and 8 %, respectively. In other words, low concentrations of BMP4 (5 to 10 ng/ml) increased the percentage of *Mvh*-positive cells in a manner consistent with other studies (1, 18, 27). Evidence exists to demonstrate that BMP4 in tissue culture is dose dependent and controls the population of PGCs (6, 20).

Epiblast cells could be differentiated into PGCs in the presence of recombinant human BMP4. The ratio of PGCs derived from epiblast cell culture increased with BMP4 (20). It has been demonstrated that treating organ cultures with BMP4 resulted in a biphasic effect on the population of PGCs. Low doses (0.5 and 5 ng/ml) increased PGCs numbers, whereas higher doses (50 and 500 ng/ml) had no effect or actually reduced PGCs (6). Wei have reported that the percentage of GFP-positive cells was higher in day four EBs supplemented with BMP4 than in EBs without BMP4. To detect whether the GFP-positive cells supplemented with BMP4 included PGCs, the expression of the PGC-related genes was tested. The expression patterns of the specific PGCs genes in GFP-positive cells induced by BMP4 were similar to the expression patterns in E7.25 PGCs (18). There have been reports that addition of BMP4 increased the expression of germ cell-specific markers Vasa (*Mvh*) during differentiation of hESCs to EBs. In contrast, expression of *Oct4* decreased in the presence and absence of BMP4. The low concentrations of BMP4 may be explained in part by the presence of different BMP receptors and signaling molecules in those cells. Indeed, studies have shown that PGCs formation in epiblast cell cultures can be induced by BMP4, and that induction is dependent on the presence of SMAD1, an intracellular signaling protein in the BMP4 signaling pathway (28).

Previous studies have demonstrated that there are Vasa-positive and Vasa-negative cells in EBs, but the higher percentage belongs to Vasa-negative cells. Thus, some of these cells were either undifferentiated or differentiated to other cells (15, 18). Our results have shown that *Mvh*-positive cells constituted a small population of cells within differentiating EBs (9.54 %), but the majority of ES cells in EBs were undifferentiated. This was consistent with an in vivo study which demonstrated that mesoderm markers were expressed in adjacent PGCs while being repressed in PGCs (15).

Thus, perhaps, only a small proportion of cells in differentiating human EBs express receptors required for establishment and/or maintenance of PGCs in ES cells (15, 20).

Also Vasa-positive cells were most frequently localized to the edge of EBs (15, 24). This bears some similarity to the specification and subsequent expansion of PGCs populations in vivo. Induction of PGCs formation in the mouse is location dependent, such that only proximal epiblast cells can be induced: distal epiblast cells do not contain key SMADs required for PGCs induction. Thus, it may be that only the cells at the edges of the EBs contain the key SMADs or other signaling components required for response (6, 15).

## Conclusion

Taken together, these results indicate that BMP4 triggers PGCs derivation by providing a favorable microenvironment in an *in vitro* culture, Moreover, EBs provide a suitable environment which is similar to an in vivo environment for the differentiation of PGCs. Thus a low concentration of BMP4 may establish a proper niche for PGCs specification and development. Low concentrations of BMP4 are considered to be functional concentrations of BMP4 in the differentiation of mESC to PGCs in vitro.
